# A Proteomic Approach Reveals That miR-423-5p Modulates Glucidic and Amino Acid Metabolism in Prostate Cancer Cells

**DOI:** 10.3390/ijms24010617

**Published:** 2022-12-29

**Authors:** Amalia Luce, Angela Lombardi, Carmela Ferri, Silvia Zappavigna, Madhura S. Tathode, Amanda K. Miles, David J. Boocock, Jayakumar Vadakekolathu, Marco Bocchetti, Roberto Alfano, Rossella Sperlongano, Angela Ragone, Luigi Sapio, Vincenzo Desiderio, Silvio Naviglio, Tarik Regad, Michele Caraglia

**Affiliations:** 1 Department of Precision Medicine, University of Campania “Luigi Vanvitelli”, Via L. De Crecchio 7, 80138 Naples, Italy; 2 John van Geest Cancer Research Centre, Nottingham Trent University, Nottingham NG11 8NS, UK; 3 Medicina Futura Group, Coleman S.p.A, Via Alcide De Gasperi 107/109/111, Acerra, 80011 Naples, Italy; 4 Laboratory of Precision and Molecular Oncology, Biogem Scarl, Institute of Genetic Research, Contrada Camporeale, 83031 Ariano Irpino, Italy; 5 Department of Advanced Medical and Surgical Sciences “DAMSS”, University of Campania “Luigi Vanvitelli”, Via S. M. di Costantinopoli 104, 80138 Naples, Italy; 6 Department of Experimental Medicine, University of Campania “Luigi Vanvitelli”, 80138 Naples, Italy

**Keywords:** microRNA, LNCaP, MALAT1, non-coding RNA, prostate adenocarcinoma, proteomics, metabolism, target genes, microtubule-associated protein 1B, overall survival

## Abstract

Recently, we have demonstrated that miR-423-5p modulates the growth and metastases of prostate cancer (PCa) cells both in vitro and in vivo. Here, we have studied the effects of miR-423-5p on the proteomic profile in order to identify its intracellular targets and the affected pathways. Applying a quantitative proteomic approach, we analyzed the effects on the protein expression profile of miR-423-5p-transduced PCa cells. Moreover, a computational analysis of predicted targets of miR-423-5p was carried out by using several target prediction tools. Proteomic analysis showed that 63 proteins were differentially expressed in miR-423-5-p-transfected LNCaP cells if compared to controls. Pathway enrichment analysis revealed that stable overexpression of miR-423-5p in LNCaP PCa cells induced inhibition of glycolysis and the metabolism of several amino acids and a parallel downregulation of proteins involved in transcription and hypoxia, the immune response through Th17-derived cytokines, inflammation via amphorin signaling, and ion transport. Moreover, upregulated proteins were related to the S phase of cell cycle, chromatin modifications, apoptosis, blood coagulation, and calcium transport. We identified seven proteins commonly represented in miR-423-5p targets and differentially expressed proteins (DEPs) and analyzed their expression and influence on the survival of PCa patients from publicly accessible datasets. Overall, our findings suggest that miR-423-5p induces alterations in glucose and amino acid metabolism in PCa cells paralleled by modulation of several tumor-associated processes.

## 1. Introduction

Prostate cancer (PCa) is one the most common cancers worldwide in males [1,2]. Despite screening based on the prostate-specific antigen (PSA), the diagnosis and treatment of PCa are frequently delayed because specific symptoms and biomarkers are lacking in the early stage [3]. Consequently, it is crucial to understand the molecular bases of PCa in order to develop novel strategies for both diagnosis and treatment.

MicroRNAs (miRNAs) are small non-coding RNAs highly conserved during evolution and key regulators of numerous genes involved in physiological processes and diseases including cancer. They can modulate several biological mechanisms by post-transcriptional inhibition of gene expression affecting downstream proteins and, therefore, cancer-related pathways. By interacting with 3′ untranslated regions (3′UTRs) of mRNA targets, miRNAs can play important regulatory roles as either oncogenes or tumor suppressors. In addition, the role of miRNAs is not limited to cancer cells, they can also act on immune, endothelial and stromal cells in the tumor tissue microenvironment, thus modulating tumor development, progression and spreading [4,5,6].

Although earlier studies showed a strong correlation between miRNAs and PCa, the investigation of new miRNAs with therapeutic and diagnostic potential is still ongoing [7,8].

Several studies revealed the double function of miR-423-5p in malignancies. miR-423-5p has been linked to cell invasion and migration in lung cancer, brain metastases, malignant phenotype and chemoresistance in glioblastoma, and cell growth in gastric cancer [9,10,11,12,13]. Conversely, it can inhibit cancer proliferation and invasion in ovarian cancer as well as colon cancer progression by the activation of caspase-dependent apoptosis [14,15].

Finally, we have recently shown that miR-423-5p plays a tumor suppressive role by inhibiting the long non-coding RNA (lncRNA) MALAT1-mediated proliferation, migration, and invasion of PCa cells. Gene expression analysis of cancer progression in PCa models overexpressing miR-423-5p demonstrated a strong reduction of both metastatic and angiogenesis processes. Moreover, these data were strongly supported by a reduction in the proliferation and metastatization induced by miR-423-5p overexpression in xenografted NOD/SCID mice [16].

Based on these results and in order to investigate the protein targets of miR-423-5p in PCa cells, we applied a quantitative proteomic approach to analyze the downstream effects on the protein expression profile of miR-423-5p-transduced PCa cells. Moreover, a computational analysis of predicted target genes of miR-423-5p was carried out by using several target prediction tools including DIANA microT-CDS, TargetScan_Human_v8.0, mirDIP v4.1, and mirDB. Results from proteomic analysis were integrated with prediction analysis and previously validated miR-423-5p targets have been additionally characterized by Gene Expression Profiling Interactive Analysis (GEPIA) and Human Protein Atlas online tools to evaluate the impact of the miR-423-5p on protein expression and clinical progression in PCa.

## 2. Results

### 2.1. Proteomic Profiling of LNCaP Cells Overexpressing miR-423-5p

To investigate the effect of miR-423-5p on its intracellular targets, we performed a quantitative SWATH proteomic analysis of PCa LNCaP cells, both those expressing miR-423-5p mimic and the control transduced with the empty vector.

**Figure 1:**
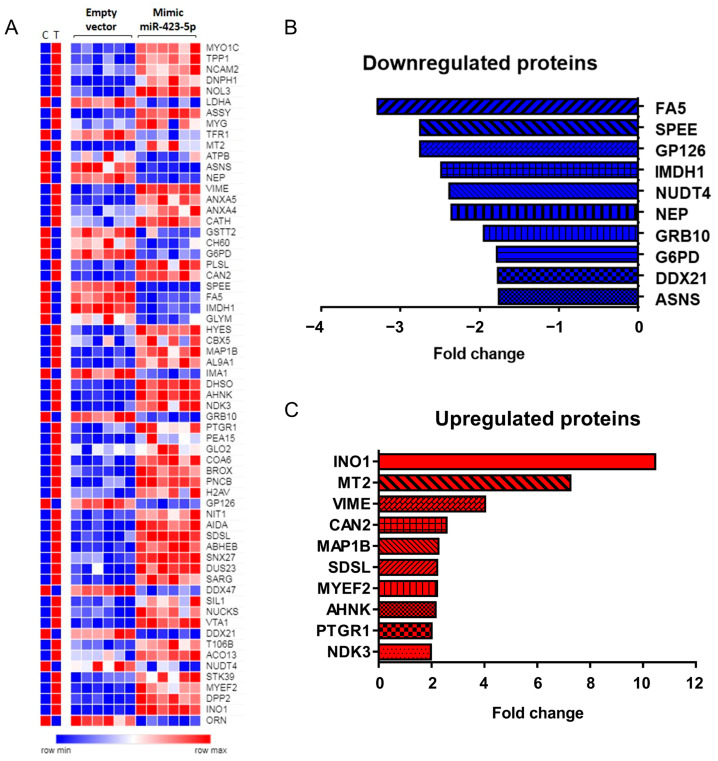
Differentially expressed proteins (DEPs) in LNCaP PCa cells expressing miR-423-5p mimic compared to control (empty vector) evaluated by quantitative SWATH-mass spectrometry and defined downregulated if fold change (FC) ≤ −1.5, and upregulated if fold change (FC) ≥ 1.5. Data analysis performed with the SCIEX OneOmics cloud processing platform (n = 6 biological replicates, OneOmics confidence over 75%). (**A**) Heatmap representation of the average of proteomic data (C = ctr, empty vector; T = treated, mimic miR-423-5p) and heatmap of the individual replicates (empty vector and mimic miR-423-5p). (**B**) Histogram graph of the top ten downregulated proteins. (**C**) Histogram graph of the top ten upregulated proteins.

Differential expression analysis showed that 63 proteins were significantly differentially expressed in the LNCaP cells overexpressing miR-423-5p compared to control, of which 19 proteins were downregulated and 44 upregulated (Figure 1A–C). A complete list of differentially expressed proteins (DEPs) is available in Table S1.

**Figure 2:**
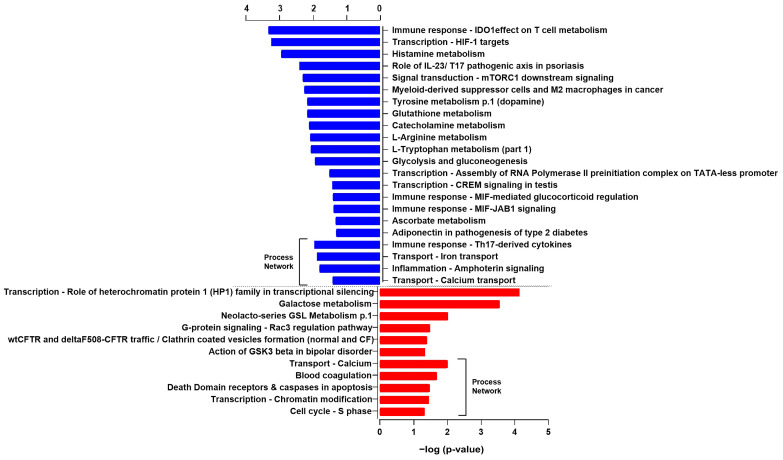
MetaCore enrichment analysis of proteomics data in LNCaP PCa cells overexpressing miR-423-5p. Significant pathways and process networks from downregulated (red) and upregulated (blue) proteins in LNCaP PCa cells expressing miR-423-5p mimic compared to control (empty vector). Quantitative SWATH-mass spectrometry data analysis performed with the SCIEX OneOmics cloud processing platform (n = 6 biological replicates).

**Figure 3:**
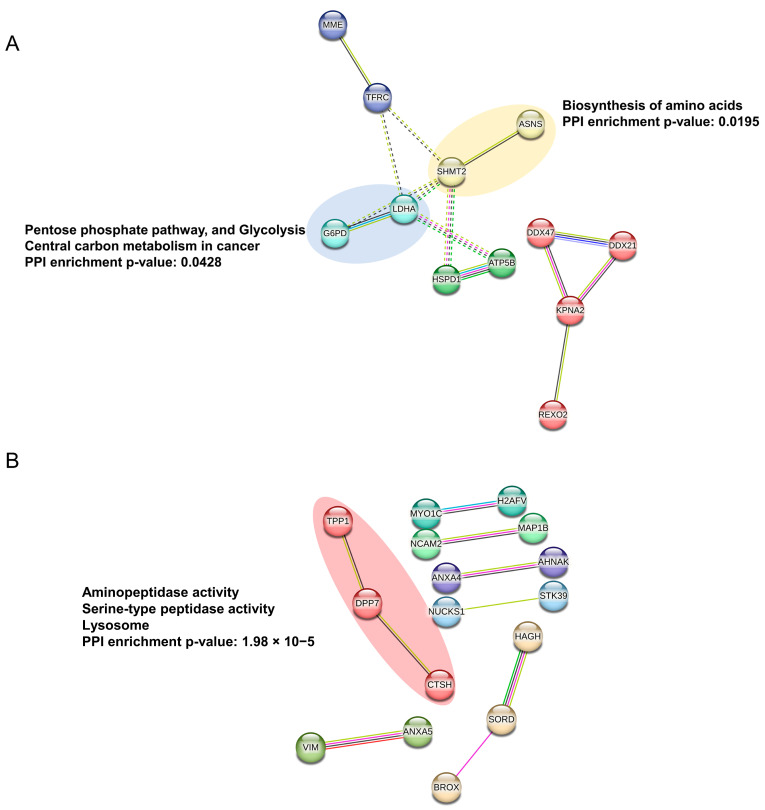
STRING analysis of proteomics data. Protein–protein interaction (PPI) networks of significant downregulated (**A**) and upregulated (**B**) proteins in LNCaP PCa cells expressing miR-423-5p mimic compared to control (empty vector). Analysis was performed with the search tool for retrieval of interacting genes/proteins database (STRING, version V11.0, [17]). Available online: http://stringdb.org, (accessed on 19 October 2022). Medium confidence was applied for the analysis and the Markov Cluster Algorithm (MCA) performed to extract clusters of densely connected nodes from biological networks.

Pathway enrichment analysis of all up- and down-regulated proteins using the online software MetaCore (Thomson Reuters) identified several pathways, (Table 1 and Table 2) modulated following miR-423-5p mimic expression. We found that most of the proteins, whose expression was downregulated, are mainly involved in cellular metabolism, such as IDO1 (Indoleamine 2,3-dioxygenase-1) effect on T cell metabolism, glycolysis and gluconeogenesis, and histamine, glutathione, tyrosine, catecholamine, and ascorbate metabolisms (Figure 2). In fact, we observed a decreased expression of G6PD (Glucose-6-phosphate dehydrogenase), HX2 (Hexokinase 2), ALDH2 (Aldehyde dehydrogenase 2 family), and LDHA (Lactate dehydrogenase A) (Table 1, Figure 2). Other downregulated proteins are involved in immune response, related to Myeloid-derived suppressor cells and M2 macrophages in cancer, MIF-mediated glucocorticoid regulation, and MIF-JAB signaling. Proteins involved in transcription and hypoxia, such as HIF-1 targets’ pathways and transcriptional assembly, were also significantly downregulated (Table 1, Figure 2). Conversely, proteins whose expression was upregulated were mostly associated with cell metabolism, such as galactose metabolism, neolacto-series GSL (Glycosphingolipid) metabolism, peptidase activity, and the lysosomal pathway but also with clathrin vesicles formation, G-protein signaling, and the HP1 role in transcriptional silencing (Table 2, Figure 2). Furthermore, when both downregulated and upregulated proteins were analyzed as process networks, those involved in the immune response through Th17-derived cytokines, inflammation via amphorin signaling, and ion transport were downregulated. Conversely, those related to the cell cycle S phase, chromatin modifications, apoptosis, blood coagulation, and calcium transport were upregulated (Table 3 and Table 4, Figure 2). Although several pathways and processes were identified in response to miR-423-5p overexpression, cellular metabolism, immune response, and transcription appear to be the ones that are mainly affected as indicated by the pathway enrichment analysis of proteomic data (*p*-value ≤ 0.05). Protein–protein interaction (PPI) networks of significant downregulated and upregulated proteins in LNCaP PCa cells expressing miR-423-5p mimic compared to control (empty vector) made by STRING analysis of proteomic data confirmed Metacore enrichment analysis [17]. As shown in Figure 3A, the majority of the proteins downregulated by miR-423-5p are involved in the pentose phosphate pathway, glycolysis, and carbon metabolism in cancer (G6PH and LDHA), as well as the biosynthesis of amino acids (SHMT2 and ASNS). Conversely, upregulated proteins in LNCaP PCa cells overexpressing miR-423-5p are involved in catabolic mechanisms related to amino acids metabolism and lysosome function (TPP1, DPP7 and, CTSH) (Figure 3B).

### 2.2. Prediction of Functional Targets by Integrative Analysis of miRNA Predicted Targets and Protein Expression Data

**Figure 4:**
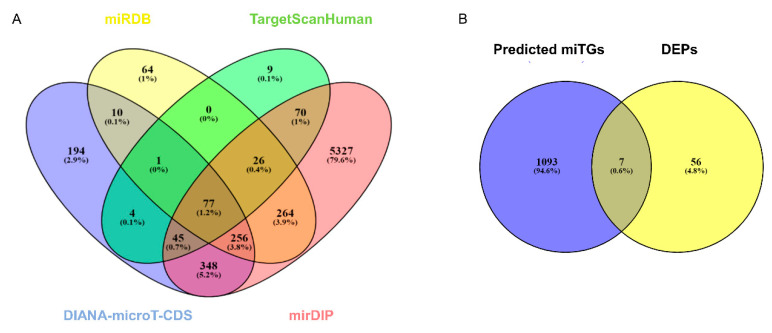
Summary of target genes of hsa-miR-423-5p (miTGs). (**A**) Venn diagram to identify common targets predicted by DIANA microT-CDS, TargetScan_Human_v8.0, mirDIP, and mirDB. (**B**) Venn diagram showing the targets commonly represented between DEPs and predicted miTGs.

Previous studies show that miR-423-5p is involved in the downregulation of lncRNA MALAT1 and promotes cell proliferation, migration, and invasion in PCa cells [16]. Moreover, the overexpression of this miRNA in PCa cells is associated with increased survival of PCa patients and reduced metastasis formation in mouse models [16]. To evaluate the targets involved in the role played by miR-423-5p in PCa, a target prediction analysis was performed. By using four target prediction tools, a total of 6695 miRNA target genes (miTGs) were identified of which 77 (1.2%) were represented in all databases (Table 5, Figure 4A) [18,19,20,21]. From the output list, miRNA targets predicted by two or more prediction tools were considered for further analysis. Then, an integrated analysis between predicted miRNA targets and DEPs identified in this study was performed. As shown in the Venn diagram, seven targets were found commonly represented between miR-423-5p target genes and DEPs in LNCaP cells overexpressing miR-423-5p (Figure 4B). A detailed summary of the seven genes/proteins commonly represented in the prediction analysis results and proteomic data is given in Table 6.

### 2.3. Protein Expression Analysis and Gene Expression Profiling of miR-423-5p Targets in Prostate Cancer

To understand the known experimental interaction between miR-423-5p and the seven validated differentially expressed target proteins from the proteomic analysis, the DIANA TarBase v8 database was used [22]. TarBase analysis showed that there is a known interaction between miR-423-5p and five out of the seven targets, namely, Microtubule-associated protein 1B (MAP1B), Chromobox 5 (CBX5/HP1A), Inosine-5′-monophosphate dehydrogenase 1 (IMPDH1), Adhesion G-protein coupled receptor G6 (ADGRG6), and Spermidine synthase (SRM), and reported at different tissue levels including bone marrow, kidney, and cervix at different experimental conditions. However, there was no known experimentally validated interaction reported between miR-423-5p and the abovementioned target genes in PCa (Table S2) [23,24,25,26,27].

To exploit the expression of protein targets of miR-423-5p in the LNCaP PCa cell line, we performed a protein expression analysis using the Expression Atlas for the previously identified seven targets in the LNCaP cell line [28]. All protein targets showed a high expression in the LNCaP cell line (High: >1.000 Parts per billion) as reported in Table 7.

Moreover, in order to extend the analysis to data from patients, a gene expression analysis was performed using GEPIA2 based on The Cancer Genome Atlas-Prostate Adenocarcinoma (TCGA-PRAD) and the Genotype-Tissue Expression (GTEx) project data [29].

**Figure 5:**
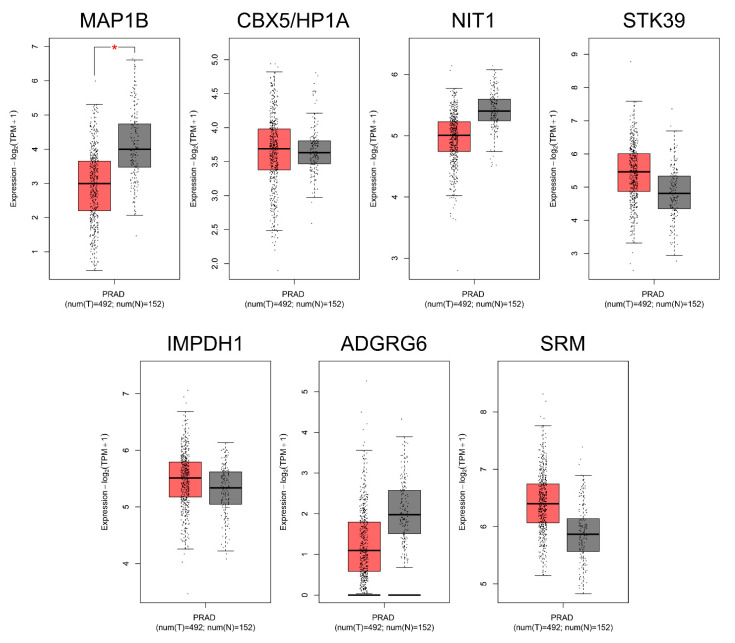
Gene expression analysis performed with GEPIA2 for the predicted miTGs in prostate adenocarcinoma (PRAD) samples (red, T = 492) and normal tissue (grey, N = 152) from TCGA and GTEx projects. Gene expression (log2(TPM + 1)) for MAP1B, CBX5/HP1A, NIT1, STK39, IMPDH1, ADGRG6 and SRM. Significant difference shown with an asterisk: *  *p*-value ≤ 0.01.

Comparison of mRNA expression in prostate adenocarcinoma (Tumour, T = 492) versus normal prostate tissue (Normal, N = 152) showed a significant difference for microtubule-associated protein 1B (MAP1B). Conversely, the other evaluated and validated targets CBX5/HP1A, NIT1, STK39, IMPDH1, ADGRG6, and SRM did not show any significant differences (Figure 5).

Finally, the analysis of the expression of IMDH1 (gene IMDPH1) and SPEE (gene SRM), which were downregulated in LNCaP cells overexpressing miR-423-5p from the proteomic analysis, showed an opposite trend at the transcriptomic level using the TCGA-PRAD and GTEx datasets, suggesting that the lowered expression of these target proteins is mediated by the overexpression of miR-423-5p in PCa cells (Table 7). This evidence is supported by the real-time expression profiling (RT-qPCR) of miR-423-5p in several human PCa cell lines (Figure S1). In fact, we observed a lower expression of miR-423-5p in LNCaP cells that was at the limit of detection if compared to DU-145 and PC-3 cells. Moreover, to determine the prognostic significance of miR-423-5p, an overall survival analysis using the ENCORI Pan-Cancer platform was performed [30]. Two clinical subgroups that consisted of patients with high and low levels of miR-423-5p were obtained but no significant correlations between the expression levels of miR-423-5p and prognosis in PRAD groups was found (log-rank *p* = 0.96) (Figure S2).

### 2.4. Overall Survival Analysis Based on the Expression of miR-423-5p Target Genes 

**Figure 6:**
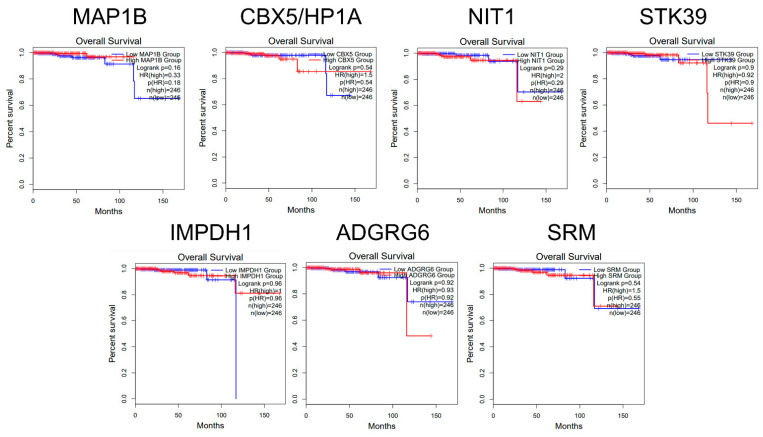
Kaplan–Meier plots for mRNA expression derived from the online GEPIA2 tool for sets of high (red) and low (blue) expression level cohorts. ‘N’ represents the size of the patient cohorts involved in the study with high and low expression of mir-423-5p targets.

To evaluate the clinical significance of the modulation of the miRNA targets in PCa, we conducted a Kaplan–Meier analysis of the overall survival using the GEPIA2 online tool [29]. Cohorts of 246 patients were assigned to low and high expression levels of mRNA for the targets found differentially expressed and no significant differences were detected in the overall survival for these targets (Figure 6).

## 3. Discussion

In the present manuscript, we investigated downstream pathways that are affected by miR-423-5p expression using mass-spectrometry analysis. We found that cellular metabolism, amino acid metabolism, and transcription are the main affected pathways. IDO1 (Indoleamine 2,3-dioxygenase-1) effect on T cell metabolism, glycolysis and gluconeogenesis, and histamine, glutathione, tyrosine, catecholamine, and ascorbate metabolisms, were the metabolic pathways affected by downregulated proteins. In these pathways, a decreased expression of G6PD, HX2, ALDH2, and LDHA was found. Other downregulated proteins are associated with immune response, including those related to Myeloid-derived suppressor cells and M2 macrophages in cancer, MIF-mediated glucocorticoid regulation, and MIF-JAB signaling. Upregulated proteins were mostly associated with cell metabolism, such as galactose metabolism, neolacto-series GSL (Glycosphingolipid) metabolism, peptidase activity, and the lysosomal pathway but also with clathrin vesicles formation, G-protein signaling, and the HP1 role in transcriptional silencing. miR-423-5p’s role in the regulation of metabolic pathways is still under-investigated. We provide for the first time, at least to our knowledge, that miR-423-5p regulates cellular metabolism in cancer cells. However, it was reported that miR-423-5p induces inhibition of neoglucogenesis, hyperglycemia, and liver deposition in an animal model of diabetes [31]. These data were confirmed in a model of gestational diabetes in which the repression of miR-423-5p ameliorated hepatic gluconeogenesis and insulin resistance [32]. G6PD, another target of miR-423-5p involved in the pentose phosphate pathway (PPP) and glucose metabolisms, was also downregulated in a model of preeclampsia and gestational hypertension [33]. In cancers, PPP has been upregulated if compared to normal counterparts becoming a possible target for therapy [34,35,36,37,38]. The involvement of G6PD in drug resistance in patients affected by acute myeloid leukemia was reported by Gregory et al. and, more recently, similar results were found in patients with multiple myeloma [39,40]. Interestingly, we have previously reported that G6PDH inhibition blocks proliferation and metastases of head and neck squamous cancer cells in vivo, and this effect was paralleled by enhanced autophagy [41,42]. These effects are in agreement with our findings about the involvement of miR-423-5p in the autophagic processes in hepatocellular cancer cell lines [43]. Conversely, no previous reports exist on the role of miR-423-5p in the regulation of amino acid metabolism. The HIF-1 pathway was also downregulated by miR-423-5p. This result is again in agreement with our previously reported findings on the anti-angiogenic effects of miR-423-5p in PCa cell lines [16]. In fact, in PCa cell lines, we have found a strong downregulation of both vascular endothelial growth factor (VEGF) B and C that are, in turn, induced by the HIF-1 pathway. It has also to be considered that miR-423-5p was reported to have pro-apoptotic effects in both cardiocytes and renal proximal tubular epithelial cells during hypoxia/reoxygenation, confirming its role in the regulation of cellular hypoxic homeostasis [44,45]. Although miR-423-5p involvement in the transcriptional silencing of HP1A is highly likely due to its post-transcriptional regulatory roles, its role in clathrin vesicle formation, and G-protein signaling, is not known, thus, requiring further investigations. We have also found an increase in the activity of lysosome formation. In this light, we have previously reported that the overexpression of miR-423-5p in hepatocellular cancer cells induced an increased activation of the autophagic process with the involvement of lysosome and the formation of abundant autophagic vacuoles [43]. Among the protein targets of miR-423-5p, we have found SPEE, which is involved in both amino acid metabolism and cancer [46]. In fact, spermidine has a role in the regulation of cancer cell proliferation and is involved in anti-cancer immune surveillance [46]. Other protein targets were MAP1B, CBX5, NIT1, STK39, IMDH1, and AGRG6. Interestingly, five out of the seven targets, namely, Micro-tubule-associated protein 1B (MAP1B), Chromobox protein homolog 5 (CBX5/HP1A), Inosine-5′-monophosphate dehydrogenase 1 (IMPDH1), Adhesion G-protein coupled receptor G6 (ADGRG6), and Spermidine synthase (SRM), are reported at different tissue levels, including cervix, kidney and bone marrow, at different experimental conditions. Integrating the results from the proteomic analysis in LNCaP cells and the gene expression profiling for the miR-423-5p targets in PRAD, we found that IMDPH1 and SRM showed an opposite trend at transcriptomic level, indicating that miR-423-5p could interact on these miTGs in PCa. Since we previously reported that the overexpression of miR-423-5p was able to inhibit MALAT1-mediated proliferation, migration, and invasion of PCa cells, we evaluated the clinical significance of miR-423-5p in the TCGA-PRAD dataset using the ENCORI Pan-Cancer analysis platform and no significant correlations between the expression level of miR-423-5p and prognosis in PRAD groups was detected [16]. Furthermore, the analysis of miTGs with GEPIA2 based on The Cancer Genome Atlas-Prostate Adenocarcinoma (TCGA-PRAD) and the Genotype-Tissue Expression (GTEx) project data demonstrated only for MAP1B a statistically significant higher expression in tumors if compared to normal counterpart in PCa patients. These results were likely due to the limited size of the samples analyzed and included in the publicly available dataset. There are no previous reports on MAP1B in PCa, but it was demonstrated to be associated with several processes in other cancers. MAPs are proteins that participate in the organization of cell cytoskeletons taking part in the microtubule assembly that is essential for the maintenance of cellular structure during cell division [47,48]. MAPs have been also correlated to microtubule dynamics required for cell motility and metastatization [49]. MAP1B was expressed to different extents in multiple cancers with a higher degree in central nervous system tumors and its levels were found to predict shorter survival and higher grade in urothelial cancers [50].

## 4. Materials and Methods

### 4.1. Cell Lines and Growth Conditions

LNCaP (ATCC^®^ CRL-1740™), DU-145 (ATCC^®^ HTB-81™) and PC-3 (ATCC^®^ CRL-1435™) PCa cell lines were purchased from ATCC as previously reported [16]. LNCaP cells were cultured in RPMI-1640 (11530586, Gibco Life Technologies, Carlsbad, CA, USA), DU-145 cells in EMEM (BE12-6621, Lonza, Basel, Switzerland) and PC-3 cells in F-12 k Nut Mix (1×) (21127-022, Gibco Life Technologies, Carlsbad, CA, USA). The media were supplemented with 10% (*v/v*) foetal calf serum (FCS) and 1% (*w/v*) L-glutamine (Lonza, Basel, Switzerland). Cells were incubated at 37 °C in 5% (*v/v*) CO_2_ and 100% (*v/v*) humidity.

### 4.2. Reverse Transcription Quantitative Real-Time PCR (RT-qPCR)

RNA was extracted from PC-3, DU-145 and LNCaP PCa cell lines using RNeasy Mini Kit (74106, Qiagen, Hilden, Germany) according to the manufacturer protocol. TaqMan Micro RNA Reverse Transcription Kit (4366596, Applied Biosystems, Waltham, MA, USA) was used to obtain cDNA using specific reverse transcription primers for miR-423-5p and U6 as housekeeping gene (4427975, Applied Biosystems, Waltham, MA, USA). RT-qPCR was performed using TaqMan Universal PCR Master Mix (4304437, Applied Biosystems, Waltham, MA, USA) and specific qPCR primers for miR-423-5p and U6 (4427975, Applied Biosystems, Waltham, MA, USA) were used. The results were analyzed using 2^–∆∆Ct^ method and data are shown as mean ± SD.

### 4.3. Retroviral Expression of Empty Vector and miR-423-5p-Mimic

LNCaP PCa cells were seeded in a 96-well plate, 35,000 cells/well, and treated with Sh MIMIC Lenti miR-423-5p lentiviral particles (V1SMHS_000254, GE Healthcare Dharmacon, Lafayette, CO, USA) at 2.5, 5, 10, and 20 MOI. Control empty backbone cells were obtained using lentiviral particles generated from the pLKO.1 Empty plasmid (MISSION^®^ pLKO.1-puro Empty Vector Control Plasmid DNA, SHC001, Sigma Aldrich, St. Louis, MO, USA). Hexadimethrine Bromide (H9268-5G, Sigma Aldrich, St. Louis, MO, USA) was used in complete growth media for each cell line to facilitate the infection. After 16 h incubation, infection media was removed and replaced with complete growth medium for each cell line. Puromycin (Invivogen, San Diego, CA, USA) was then added to each cell’s complete growth medium at a final concentration of 1 µg/mL for the selection. The lentiviral particles used to infect LNCaP were produced according to the manufacturer’s recommendations. The lentiviral packaging mix was purchased from Sigma (MISSION^®^ Lentiviral Packaging Mix, SHP001, Sigma Aldrich, St. Louis, MO, USA). MOI of 5 was selected for the following experiments.

### 4.4. Liquid Chromatography with Tandem Mass Spectrometry (LC-MS/MS) Analysis

Samples (~50 µg protein) were reduced/alkylated and digested as described previously [51]. Then, samples were dried and resuspended in 5% (*v/v*) acetonitrile + 0.1% (*v/v*) formic acid and transferred to an HPLC vial for MS analysis in both SWATH (Sequential Window Acquisition of all Theoretical Mass Spectra) and IDA (information-dependent acquisition) modes. Each sample was analyzed on a SCIEX TripleTof 6600 mass spectrometer coupled in line with an Eksigent ekspert nano LC 425 system running in microflow as described previously with minor modifications [52]. For IDA (Information-Dependent Analysis) to generate a spectral library, 8 μL of pooled sample in triplicate were injected by autosampler (Eksigent nanoLC 425 LC system, Dublin, OH, USA) in microflow at 5 μL/min directly onto a YMC Triart-C18 column (15 cm, 3 μm, 300 μm i.d.) using gradient elution (2–40% Mobile phase B, followed by washing at 80% B and re-equilibration) over 87 min. For SWATH/DIA (Data-Independent Analysis), 3 μL was injected on the same gradient elution profile over 57 min. Mobile phases consisted of A: 0.1% formic acid; B: acetonitrile containing 0.1% (*v/v*) formic acid. IDA analysis was carried out in positive ion mode with a 250 ms survey scan, *m*/*z* range 400–1250; Top 30 peaks selected for fragmentation, accumulation time 50 ms per experiment, cycle time 1.8 s. SWATH analysis used 100 variable windows, 25 ms per window, 100–1500 *m*/*z* using the SCIEX Duospray source with a 50 μm electrode at 5500 V.

### 4.5. Library Generation, Spectral Alignment, and Fold Change Analysis

Spectral libraries were constructed from three IDA runs of pooled samples spiked with HRM-kit retention time peptides (iRT, Biognosys AG, Schlieren, Switzerland), searched using ProteinPilot 5.0 (SCIEX, Macclesfield, UK) against the Swissprot human database (January 2016). The resulting ion library file was aligned against the iRT peptides, and the SWATH data was obtained using the OneOmics cloud processing platform (SCIEX, Warrington, UK) with the following parameters: six peptides per protein, six transitions per peptide, XIC width set at 75 ppm, 5 min extraction window. Analysis of six biological replicates per group was carried out in Protein Expression Workflow within OneOmics to identify significantly differentially expressed proteins, and a 75% confidence limit and a minimum of two peptides per protein were used as criteria for assessing fold change. Differentially expressed proteins (DEPs) were defined as downregulated if fold change (FC) ≤ −1.5. and upregulated if fold change (FC) ≥ 1.5.

### 4.6. Bioinformatic Analysis

The pathway enrichment analysis on proteomics data was performed using METACORE (version 6.34.69200, Clarivate Analytics, London, UK), available online: https://portal.genego.com (accessed on 11 April 2018). Protein–protein interaction (PPI) network analysis was performed with the search tool for retrieval of interacting genes/proteins database (STRING, version V11.0), available online: http://stringdb.org (accessed on 19 October 2022). Medium confidence was applied for the analysis and the Markov Cluster Algorithm (MCA) performed to extract clusters of densely connected nodes from biological networks.

Target prediction analysis of miRNA hsa-miR-423-5p was performed using four different prediction tools: DIANA microT-CDS Version 5.0, available online: https://dianalab.e-ce.uth.gr/html/dianauniverse/index.php?r=microT_CDS (accessed on 25 September 2022), TargetScan_Human Release v8.0, available online: https://www.targetscan.org/vert_80/ (accessed on 25 September 2022), mirDIP Release v4.1, available online: https://ophid.utoronto.ca/mirDIP/ (accessed on 25 September 2022), mirDB Version v6.0, available online: http://mirdb.org/ (accessed on 25 September 2022) [18,19,20,21]. Targets predicted by two or more prediction tools were selected for further analysis.

Venny v2.1 software tool, available online: https://bioinfogp.cnb.csic.es/tools/venny/ (accessed on 25 September 2022) was used to identify common targets between the prediction analysis and proteomic analysis.

Database of experimentally supported miRNA–gene interaction tool DIANA TarBase Version 8.0, available online: https://dianalab.e-ce.uth.gr/html/diana/web/index.php?r=tarbasev8/index (accessed on 25 September 2022) was used to study known interaction between hsa-miR-423-5p and validated target proteins in existing PCa studies [22]. Expression Atlas Release 38, available online: https://www.ebi.ac.uk/gxa/home (accessed on 26 September 2022), and GEPIA2, available online: http://gepia2.cancer-pku.cn/#index (accessed on 26 September 2022) were used to study the expression pattern of validated proteins and miTGs in existing PCa studies [28,29].

Gene expression profiles of miTGs were generated using GEPIA2 with the following parameters: |Log2FC| Cutoff: 1, *p*-value Cutoff: 0.01, log scale: log2(TPM + 1) matching TCGA normal and GTEx data.

Overall Survival plots were obtained using the “Survival Analysis” module of GEPIA2 with default parameters including a 95% confidence interval, “Median” Group cut off and Hazard Ratio (calculated based on the Cox PH model) using both the TCGA-PRAD and GTEx prostate datasets.

To correlate miR-423-5p expression levels with overall survival in PCa, “Pan-Cancer” module of RNA Interactome - ENCORI (The Encyclopedia of RNA Interactomes) v2.0 platform was used. Available online: https://starbase.sysu.edu.cn/ (accessed on 19 December 2022).

## 5. Conclusions

In conclusion, our data indicate that miR-423-5p could induce a metabolic switch inhibiting essential energetic processes such as glycolysis and interfering with the biosynthetic amino acidic pathways in PCa cells. These effects are paralleled by the interference with angiogenetic processes as previously reported by us in PCa cells and in agreement with our previous reports on the autophagic pathway activation mediated by miR-423-5p in other cancer models. Further in vitro and in vivo experiments are needed in order to validate the identified miR-423-5-p targets. These data can open a scenario of intervention based on targeting of the newly identified miR-423-5p interactors.

## Figures and Tables

**Table 1:** MetaCore enrichment analysis of proteomics data in LNCaP PCa cells overexpressing miR-423-5p. Significant pathways modulated by downregulated proteins in LNCaP PCa cells expressing miR-423-5p mimic compared to control (empty vector) (*p*-value ≤0.05). Quantitative SWATH-mass spectrometry data analysis performed with the SCIEX OneOmics cloud processing platform (n = 6 biological replicates).

N°	Pathways Modulated by Downregulated Proteins	Total	In Data	*p*-Value	Network Objects from Active Data
1	Immune response_The effect of IDO1 on T cell metabolism	89	3	4.910 × 10^−4^	LDHA, HX2, G6PD
2	Transcription_HIF-1 targets	95	3	5.945 × 10^−4^	LDHA, TfR1, HX2
3	Histamine metabolism	29	2	1.185 × 10^−3^	ALDHA1, ALDH2
4	Role of IL-23/T17 pathogenic axis in psoriasis	54	2	4.067 × 10^−3^	Calgranulin A, Calgranulin B
5	Signal transduction_mTORC1 downstream signaling	61	2	5.163 × 10^−3^	GRB10, G6PD
6	Myeloid-derived suppressor cells and M2 macrophages in cancer	64	2	5.669 × 10^−3^	Calgranulin A, Calgranulin B
7	Tyrosine metabolism p.1 (dopamine)	71	2	6.937 × 10^−3^	ALDHA1, ALDH2
8	Glutathione metabolism	71	2	6.937 × 10^−3^	GSTT2, G6PD
9	Catecholamine metabolism	76	2	7.914 × 10^−3^	ALDHA1, ALDH2
10	L-Arginine metabolism	79	2	8.529 × 10^−3^	ALDHA1, ALDH2
11	L-Tryptophan metabolism (part 1)	81	2	8.950 × 10^−3^	ALDHA1, ALDH2
12	Glycolysis and gluconeogenesis	94	2	1.191 × 10^−2^	LDHA, HX2
13	Transcription_Assembly of RNA Polymerase II preinitiation complex on TATA-less promoters	18	1	3.164 × 10^−2^	AMH
14	Transcription_CREM signaling in testis	22	1	3.854 × 10^−2^	HX2
15	Immune response_MIF-mediated glucocorticoid regulation	23	1	4.026 × 10^−2^	MIF
16	Immune response_MIF-JAB1 signaling	24	1	4.197 × 10^−2^	MIF
17	Ascorbate metabolism	28	1	4.880 × 10^−2^	ALDH2
18	Adiponectin in pathogenesis of type 2 diabetes	29	1	5.050 × 10^−2^	ACADM

**Table 2:** MetaCore enrichment analysis of proteomics data in LNCaP PCa cells overexpressing miR-423-5p. Significant pathways modulated by upregulated proteins in LNCaP PCa cells expressing miR-423-5p mimic compared to control (empty vector) (*p*-value ≤ 0.05). Quantitative SWATH-mass spectrometry data analysis performed with the SCIEX OneOmics cloud processing platform (n = 6 biological replicates).

N°	Pathways Modulated by Upregulated Proteins	Total	In Data	*p*-Value	Network Objects from Active Data
1	Transcription_Role of heterochromatin protein 1 (HP1) family in transcriptional silencing	40	3	7.294 × 10^−5^	HP1 alpha, HP1 beta, HP1
2	Galactose metabolism	63	3	2.843 × 10^−4^	GALK1, GALT
3	Neolacto-series GSL Metabolism p.1	72	2	9.649 × 10^−3^	ASNA1
4	G-protein signaling_Rac3 regulation pathway	16	1	3.279 × 10^−2^	Calmyrin
5	wtCFTR and deltaF508-CFTR traffic/Clathrin coated vesicles formation (normal and CF)	20	1	4.083 × 10^−2^	Myosin I
6	Action of GSK3 beta in bipolar disorder	23	1	4.681 × 10^−2^	MAP-1B

**Table 3:** MetaCore enrichment analysis of proteomics data in LNCaP PCa cells overexpressing miR-423-5p. Significant process networks modulated by downregulated proteins in LNCaP PCa cells expressing miR-423-5p mimic compared to control (empty vector) (*p*-value ≤ 0.05). Quantitative SWATH-mass spectrometry data analysis performed with the SCIEX OneOmics cloud processing platform (n = 6 biological replicates).

N°	Process Networks Modulated by Downregulated Proteins	Total	In Data	*p*-Value	Network Objects from Active Data
1	Immune response_Th17-derived cytokines	98	2	1.128 × 10^−2^	Calgranulin A, Calgranulin B
2	Transport_Iron transport	108	2	1.359 × 10^−2^	TfR1, Junctin
3	Inflammation_Amphoterin signaling	118	2	1.608 × 10^−2^	Calgranulin A, Calgranulin B
4	Transport_Calcium transport	192	2	3.987 × 10^−2^	Calgranulin A, Junctin

**Table 4:** MetaCore enrichment analysis of proteomics data in LNCaP PCa cells overexpressing miR-423-5p. Significant process networks modulated by upregulated proteins in LNCaP PCa cells expressing miR-423-5p mimic compared to control (empty vector) (*p*-value ≤ 0.05). Quantitative SWATH-mass spectrometry data analysis performed with the SCIEX OneOmics cloud processing platform (n = 6 biological replicates).

N°	Process Networks Modulated by Upregulated Proteins	Total	In Data	*p*-Value	Network Objects from Active Data
1	Transport_Calcium	192	3	9.894 × 10^−3^	Annexin V, Calmyrin
2	Blood coagulation	94	2	2.056 × 10^−2^	Annexin V, Annexin IV
3	Apoptosis Death Domain receptors & caspases in apoptosis	123	2	3.390 × 10^−2^	PEA15
4	Transcription_Chromatin modification	127	2	3.595 × 10^−2^	HP1 alpha, HP1 beta
5	Cell cycle_S phase	149	2	4.806 × 10^−2^	HP1 alpha, HP1

**Table 5:** Target genes of hsa-miR-423-5p (miTGs) predicted by DIANA microT-CDS, TargetScan_Human_v8.0, mirDIP, and mirDB.

Prediction Tools	Predicted miTGs (miRNA Target Genes)
DIANA microT-CDS	935
TargetScan_Human_v8.0	232
mirDIP	6413
mirDB	698
Targets predicted by all 4 tools	6695
Targets predicted by a minimum 2/4 prediction tools	1100

**Table 6:** Predicted target genes of hsa-miR-423-5p (miTGs) and corresponding proteins modulated by the overexpression of miR-423-5p in LNCaP cells.

Gene Name	Protein Name	Protein Description	FC ^1^ miR-423-5p/ctr
MAP1B	MAP1B	Microtubule-associated protein 1B	2.27
CBX5/HP1A	CBX5	Chromobox protein homolog 5	1.62
NIT1	NIT1	Deaminated glutathione amidase	1.62
STK39	STK39	STE20/SPS1-related proline-alanine-rich protein kinase	1.56
IMPDH1	IMDH1	Inosine-5′-monophosphate dehydrogenase 1	−2.49
ADGRG6	AGRG6	Adhesion G-protein coupled receptor G6	−2.75
SRM	SPEE	Spermidine synthase	−2.75

^1^ FC: Fold Change obtained with Protein Expression Workflow using OneOmics Suite (Sciex).

**Table 7:** Expression status of miR-423-5p target proteins from mass spectrometry analysis, protein expression level in LNCaP cell line from Atlas, and miTGs expression pattern of prostate adenocarcinoma (PRAD) dataset obtained using GEPIA2.

Protein Name	Protein Description	Protein Expression Status LNCaP Cell Line with miR-423-5p/ctr	Protein Expression Level LNCaP Cell Line ^1^	miTGs ^2^ Name	Transcriptomic Expression Status PRAD ^3^ (T/N)
MAP1B	Microtubule-associated protein 1B	Upregulated	High	MAP1B	Downregulated
CBX5	Chromobox protein homolog 5	Upregulated	High	CBX5/HP1A	Upregulated
NIT1	Deaminated glutathione amidase	Upregulated	High	NIT1	Downregulated
STK39	STE20/SPS1-related proline-alanine-rich protein kinase	Upregulated	High	STK39	Upregulated
IMDH1	Inosine-5′-monophosphate dehydrogenase 1	Downregulated	High	IMPDH1	Upregulated
AGRG6	Adhesion G-protein coupled receptor G6	Downregulated	High	ADGRG6	Downregulated
SPEE	Spermidine Synthase	Downregulated	High	SRM	Upregulated

^1^ In Expression Atlas database protein expression values are expressed in parts per billion; ^2^ miTGs: miRNA Target Genes; ^3^ PRAD: Prostate Adenocarcinoma, datasets from TCGA and GTEx projects including T = Tumoral (prostate adenocarcinoma) and N = Normal samples (prostate tissue).

## Data Availability

Proteomics data presented in this study have been provided as a Supplementary Table (Table S1). Bioinformatic data analyzed in this study are publicly available.

## Supplementary Materials

The following supporting information can be downloaded at: https://www.mdpi.com/article/10.3390/ijms24010617/s1.
