# Supplementary material for: A Proteomic Approach Reveals That miR-423-5p Modulates Glucidic and Amino Acid Metabolism in Prostate Cancer Cells
Source: Int J Mol Sci. 2022 Dec 29;24(1):617. doi: 10.3390/ijms24010617 (PMC9820599; doi:10.3390/ijms24010617)
Supplement: Supplementary file 1 [file ijms-24-00617-s001.zip › ijms-2074075-supplementary.pdf]

Protein Name	UniProt Accession ID	Ensemble Protein ID	Description	Regulation Status	FC miR-423-5p/ ctr	Confidence
INO1	Q9NPH2	ENSP00000337746	Inositol-3-phosphate synthase 1	UPREGULATED	10.464	0.770
MT2	P02795	ENSP00000245185	Metallothionein-2	UPREGULATED	7.257	0.858
VIME	P08670	ENSP00000224237	Vimentin	UPREGULATED	4.032	0.873
CAN2	P17655	ENSP00000295006	Calpain-2 catalytic subunit	UPREGULATED	2.567	0.781
MAP1B	P46821	ENSP00000296755	Microtubule-associated protein 1B	UPREGULATED	2.265	0.880
SDSL	Q96GA7	ENSP00000341117	Serine dehydratase-like	UPREGULATED	2.218	0.789
MYEF2	Q9P2K5	ENSP00000267836	Myelin expression factor 2	UPREGULATED	2.213	0.803
AHNK	Q09666	ENSP00000257247	Neuroblast differentiation-associated protein AHNAK	UPREGULATED	2.154	0.893
PTGR1	Q14914	ENSP00000311572	Prostaglandin reductase 1	UPREGULATED	2.002	0.752
NDK3	Q13232	ENSP00000219302	Nucleoside diphosphate kinase 3	UPREGULATED	1.973	0.798
VTA1	Q9NP79	ENSP00000356593	Vacuolar protein sorting-associated protein VTA1 homolog	UPREGULATED	1.955	0.855
NCAM2	O15394	ENSP00000284894	Neural cell adhesion molecule 2	UPREGULATED	1.930	0.870
MYO1C	O00159	ENSP00000354283	Unconventional myosin-Ic	UPREGULATED	1.913	0.912
AIDA	Q96BJ3	ENSP00000339161	Axin interactor, dorsalization-associated protein	UPREGULATED	1.873	0.908
PLSL	P13796	ENSP00000315757	Plastin-2	UPREGULATED	1.871	0.869
ASSY	P00966	ENSP00000253004	Argininosuccinate synthase	UPREGULATED	1.863	0.866
DUS23	Q9BVJ7	ENSP00000357087	Dual specificity protein phosphatase 23	UPREGULATED	1.838	0.882
ABHEB	Q96IU4	ENSP00000318248	Protein ABHD14B	UPREGULATED	1.835	0.878
DPP2	Q9UHL4	ENSP00000360635	Dipeptidyl peptidase 2	UPREGULATED	1.831	0.809
AL9A1	P49189	ENSP00000346827	4-trimethylaminobutyraldehyde dehydrogenase	UPREGULATED	1.829	0.928
MYG	P02144	ENSP00000352835	Myoglobin	UPREGULATED	1.776	0.759
PNCB	Q6XQN6	ENSP00000341136	Nicotinate phosphoribosyltransferase	UPREGULATED	1.774	0.919
DNPH1	O43598	ENSP00000230431	2'-deoxynucleoside 5'-phosphate N-hydrolase 1	UPREGULATED	1.696	0.941
ACO13	Q9NPJ3	ENSP00000230048	Acyl-coenzyme A thioesterase 13	UPREGULATED	1.693	0.806
COA6	Q5JTJ3	ENSP00000355571	Cytochrome c oxidase assembly factor 6 homolog	UPREGULATED	1.679	0.910
ANXA5	P08758	ENSP00000296511	Annexin A5	UPREGULATED	1.670	0.901
BROX	Q5VW32	ENSP00000343742	BRO1 domain-containing protein BROX	UPREGULATED	1.663	0.926
HYES	P34913	ENSP00000369843	Bifunctional epoxide hydrolase 2	UPREGULATED	1.645	0.803
TPP1	O14773	ENSP00000299427	Tripeptidyl-peptidase 1	UPREGULATED	1.645	0.894
PEA15	Q15121	ENSP00000353660	Astrocytic phosphoprotein PEA-15	UPREGULATED	1.643	0.857
CATH	P09668	ENSP00000220166	Pro-cathepsin H	UPREGULATED	1.643	0.795
CBX5	P45973	ENSP00000209875	Chromobox protein homolog 5	UPREGULATED	1.616	0.868
NIT1	Q86X76	ENSP00000356986	Nitrilase homolog 1	UPREGULATED	1.616	0.800
T106B	Q9NUM4	ENSP00000379901	Transmembrane protein 106B	UPREGULATED	1.597	0.843
SIL1	Q9H173	ENSP00000265195	Nucleotide exchange factor SIL1	UPREGULATED	1.592	0.827
ANXA4	P09525	ENSP00000377833	Annexin A4	UPREGULATED	1.561	0.811
NUCKS	Q9H1E3	ENSP00000356110	Nuclear ubiquitous casein and cyclin-dependent kinase substrate 1	UPREGULATED	1.558	0.909
STK39	Q9UEW8	ENSP00000348278	STE20/SPS1-related proline-alanine-rich protein kinase	UPREGULATED	1.558	0.887
H2AV	Q71UI9	ENSP00000222690	Histone H2A.V	UPREGULATED	1.544	0.814
NOL3	O60936	ENSP00000268605	Nucleolar protein 3	UPREGULATED	1.509	0.763
GLO2	Q16775	ENSP00000380511	Hydroxyacylglutathione hydrolase, mitochondrial	UPREGULATED	1.508	0.772
DHSO	Q00796	ENSP00000267814	Sorbitol dehydrogenase	UPREGULATED	1.503	0.933
SARG	Q9BW04	ENSP00000352447	Specifically androgen-regulated gene protein	UPREGULATED	1.502	0.870
SNX27	Q96L92	ENSP00000357831	Sorting nexin-27	UPREGULATED	1.501	0.789
FA5	P12259	ENSP00000356770	Coagulation factor V	DOWNREGULATED	-3.291	0.759
SPEE	P19623	ENSP00000366156	Spermidine synthase	DOWNREGULATED	-2.753	0.926
GP126	Q86SQ4	ENSP00000230173	Adhesion G-protein coupled receptor G6	DOWNREGULATED	-2.753	0.804
IMDH1	P20839	ENSP00000265385	Inosine-5'-monophosphate dehydrogenase 1	DOWNREGULATED	-2.487	0.924
NUDT4	Q9NZJ9	ENSP00000338352	Diphosphoinositol polyphosphate phosphohydrolase 2	DOWNREGULATED	-2.385	0.897

NEP	P08473	ENSP00000353679	Neprilysin	DOWNREGULATED	-2.357	0.866
GRB10	Q13322	ENSP00000338543	Growth factor receptor-bound protein 10	DOWNREGULATED	-1.948	0.760
G6PD	P11413	ENSP00000358633	Glucose-6-phosphate 1-dehydrogenase	DOWNREGULATED	-1.785	0.885
DDX21	Q9NR30	ENSP00000346120	Nucleolar RNA helicase 2	DOWNREGULATED	-1.770	0.849
ASNS	P08243	ENSP00000175506	Asparagine synthetase [glutamine-hydrolyzing]	DOWNREGULATED	-1.760	0.951
LDHA	P00338	ENSP00000227157	L-lactate dehydrogenase A chain	DOWNREGULATED	-1.749	0.964
GSTT2	P0CG30	ENSP00000290765	Glutathione S-transferase theta-2B	DOWNREGULATED	-1.716	0.803
IMA1	P52292	ENSP00000332455	Importin subunit alpha-1	DOWNREGULATED	-1.702	0.814
ATPB	P06576	ENSP00000262030	ATP synthase subunit beta, mitochondrial	DOWNREGULATED	-1.676	0.814
TFR1	P02786	ENSP00000353224	Transferrin receptor protein 1	DOWNREGULATED	-1.643	0.904
CH60	P10809	ENSP00000340019	60 kDa heat shock protein, mitochondrial	DOWNREGULATED	-1.595	0.961
ORN	Q9Y3B8	ENSP00000265881	Oligoribonuclease, mitochondrial	DOWNREGULATED	-1.578	0.862
GLYM	P34897	ENSP00000333667	Serine hydroxymethyltransferase, mitochondrial	DOWNREGULATED	-1.544	0.870
DDX47	Q9H0S4	ENSP00000319578	Probable ATP-dependent RNA helicase DDX47	DOWNREGULATED	-1.527	0.850

Table S1. Differentially Expressed Proteins (DEPs) in PCa LNCaP cell line overexpressing miR-423-5p compared to the CTR (empty vector).

Gene name	Description	miRNA	Tissue	Cell line	Methods	Experimental Condition	Publications
MAP1B	Microtubule-associated protein 1B	hsa-miR-423-5p	Bone marrow	HMSC	Immunoprecipitation: HITS-CLIP	N/A	Balakrishnan I et al., 2014 [23]
			Kidney	HEK293S	Immunoprecipitation: HITS-CLIP	Treatment: hippuristanol	Karginov FV et al., 2013 [24]
CBX5/HP1A	Chromobox protein homolog 5	hsa-miR-423-5p	Cervix	TZM-bl	Immunoprecipitation: PAR-CLIP	shRNAs against HIV-1	Whisnant Adam W et al., 2013 [25]
NIT1	Deaminated glutathione amidase	hsa-miR-423-5p	N/A	N/A	N/A	N/A	N/A
STK39	STE20/SPS1-related proline-alanine-rich protein kinase	hsa-miR-423-5p	N/A	N/A	N/A	N/A	N/A
IMPDH1	Inosine-5'-monophosphate dehydrogenase 1	hsa-miR-423-5p	Bone Marrow	BCBL-1	Immunoprecipitation: HITS-CLIP	N/A	Haecker Irina et al., 2012 [26]
			Kidney	HEK293S	Immunoprecipitation: HITS-CLIP	no treatment (control)	Karginov FV et al., 2013 [24]
ADGRG6	Adhesion G-protein coupled receptor G6	hsa-miR-423-5p	Cervix	TZM-bl	Immunoprecipitation: PAR-CLIP	shRNAs against HIV-1	Whisnant Adam W et al., 2013 [25]
SRM	Spermidine synthase	hsa-miR-423-5p	Kidney	HEK293S	Immunoprecipitation: HITS-CLIP	no treatment (control)	Karginov FV et al., 2013 [24]
			Kidney	HEK293S	Immunoprecipitation: HITS-CLIP	treatment: arsenite	Karginov FV et al., 2013 [24]
			Cervix	TZM-bl	Immunoprecipitation: PAR-CLIP	shRNAs against HIV-1	Whisnant Adam W et al., 2013 [25]
			Cervix	HeLa	Immunoprecipitation: HITS-CLIP	HeLa cells treated with shRNA.	Xue Y et al., 2013 [27]

Table S2. TarBase analysis of experimentally supported interaction between hsa-miR-423-5p and differentially expressed targets in LNCaP PCa cells overexpressing miR-423-5p. Analysis confirms that there is no known interaction reported between hsa-miR-423-5p and seven targets in PCa tissue [23-27].

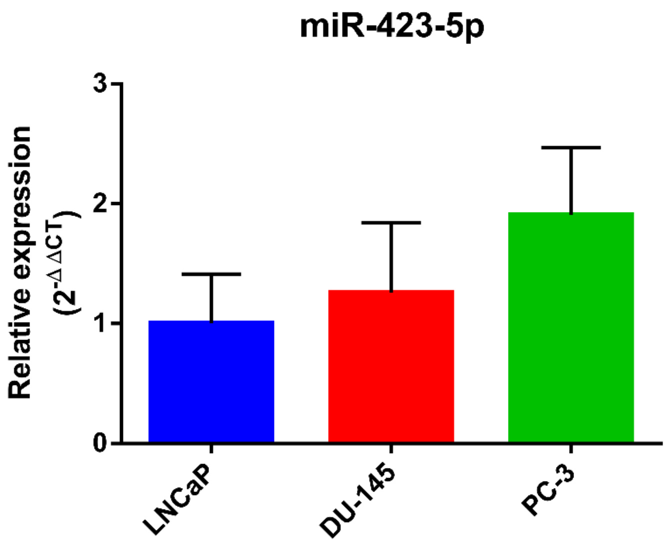

Figure S1. Analysis of miR-423-5p expression levels in human PCa cell lines by RT-qPCR. The results were analyzed using $2^{-\Delta\Delta Ct}$ method and data are shown as mean ± SD.

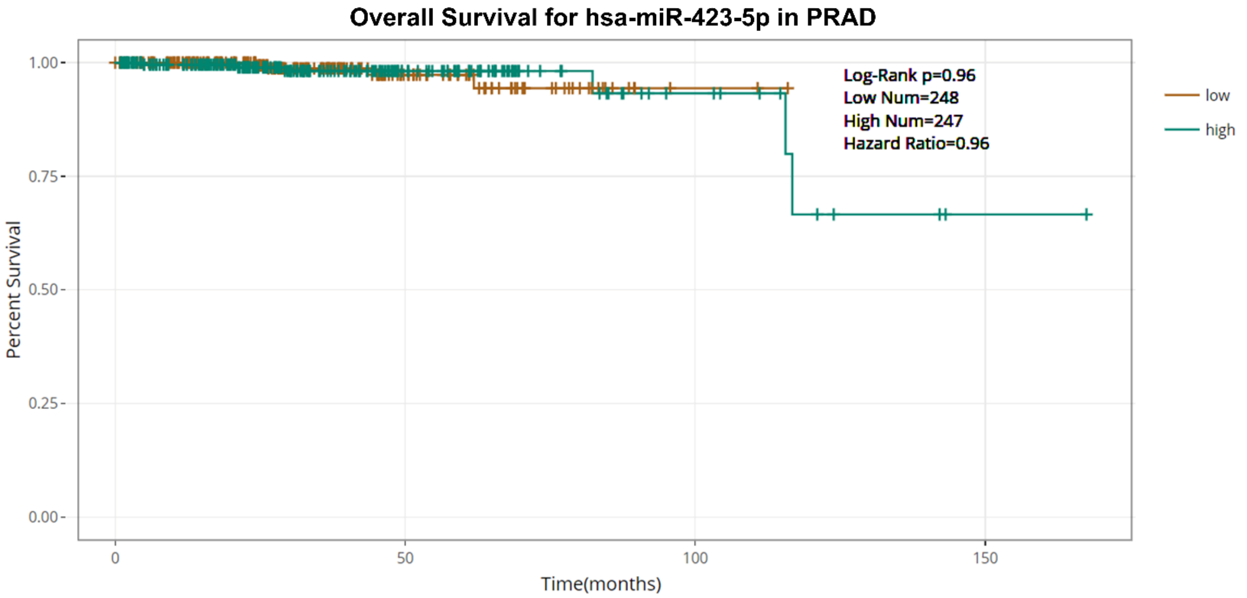

Figure S2. Correlation of miR-423-5p expression levels with overall survival in PCa. Clinical significance of miR-423-5p was evaluated using Kaplan–Meier plot analysis in PCa patients (TCGA-PRAD). Analysis was performed using "Pan-Cancer" module of RNA Interactome - ENCORI (The Encyclopedia of RNA Interactomes) v2.0 platform, available online: https://starbase.sysu.edu.cn/ (accessed on 19 December 2022) [30].
